# Evaluation of the Effect of Mineral Oil Exposure on Changes in the Structure and Mechanical Properties of Polymer Parts Produced by Additive Manufacturing Techniques

**DOI:** 10.3390/ma17153680

**Published:** 2024-07-25

**Authors:** Marcin Głowacki, Katarzyna Skórczewska, Krzysztof Lewandowski, Adam Mazurkiewicz, Piotr Szewczykowski

**Affiliations:** 1 Faculty of Mechanical Engineering, Bydgoszcz University of Science and Technology, Kaliskiego 7 Street, 85-796 Bydgoszcz, Poland; adam.mazurkiewicz@pbs.edu.pl (A.M.); piotr.szewczykowski@pbs.edu.pl (P.S.); 2 Faculty of Chemical Technology and Engineering, Bydgoszcz University of Science and Technology, Seminaryjna 3 Street, 85-326 Bydgoszcz, Poland; krzysztof.lewandowski@pbs.edu.pl

**Keywords:** 3D printing, FDM method, mineral oil, high temperature, environmental resistance, PLA, ABS, HIPS, ASA

## Abstract

The paper describes the type of changes in the structure and mechanical properties of 3D printed shapes under the influence of mineral oil. The effects of a room (23 °C) and elevated temperature (70 °C) on 3D prints manufactured by the FDM method and stored in oil for 15, 30, and 60 days on the change of properties and structure were investigated. The samples were produced from ABS (poly(acrylonitrile-co-butadiene-co-styrene)), ASA (poly(acrylonitrile-co-styrene-co-acrylate), PLA (poly(lactic acid)), and HIPS (high-impact polystyrene). Tests related to the strength of the materials, such as the static tensile test and Charpy impact test, were carried out. The structure was evaluated using a scanning electron microscope, and changes in chemical structure were determined by conducting FTIR (Fourier transform infrared spectroscopy) and TGA (thermogravimetric analysis) tests. The analysis of the results provided important information about the impact of mineral oil on specific materials. This is critical for designing and manufacturing components that can withstand mineral oil exposure in real-world environments. The materials underwent varying changes. Strength increased for PLA by about 28%, remained unchanged for ABS and HIPS during exposure for 30 days, and decreased for ASA with extended exposure up to 14%.

## 1. Introduction

Three-dimensional printing is a relatively new technology for the manufacturing industry. Its use has several advantages related to material variety and the possibility of rapid prototyping and manufacturing [1,2]. Fused Deposition Modeling (FDM) printing technology is the most commonly used method for manufacturing objects [3]. Applications of FDM technology can be found in aerospace-related industries and the medical industry [4]. The effect of the widespread use of additive printing technology is that the products manufactured by this method appear in almost every field of technology. Researchers are not only studying the available materials on the market but also creating modifications to the base materials—for example, modifications of PLA with amorphous polyhydroxyalkanoates (APHAs) derived from food waste [5]. Therefore, it is necessary to have a thorough understanding of the phenomena occurring in the degradation of printed components used not in the laboratory, but in real conditions. A better understanding of the phenomena occurring during the life of a product will help improve the design process and the selection of appropriate materials for specific applications. There is little information on the effects of varying environmental factors on 3D prints, which significantly differ in behavior from parts manufactured using conventional processing methods. Therefore, gaining information on the effect of MO as a widely existing factor in the industrial environment on the properties of 3D prints is an important contribution to this area of knowledge.

The materials used in the experiment, such as ABS (poly(acrylonitrile-co-butadiene-co-styrene) and HIPS (high-impact polystyrene), have an amorphous structure, making them strong and durable. They may, as a result of prolonged exposure to MO, tend to interact with and absorb oil [6]. ASA (poly(acrylonitrile-co-styrene-co-acrylate), a material with a modified structure similar to ABS, is a weather and UV-resistant material. Despite its high strength, it can lose its durability and resistance. On the other hand, PLA (poly(lactic acid)), as a biodegradable material obtained from lactic acid with a chain structure, can soften or swell in contact with MO, leading to reduced mechanical strength and changes in geometric dimensions [7,8]. Components made from these materials are used in many fields, ranging from prototyping to the production of final details such as gears and various types of covers and housings. They should perform their intended functions and maintain quality in a variety of environments, but a thorough understanding is necessary of how various external factors present in their working environment affect their strength.

According to descriptions in the literature, liquids such as salt and sugar solution [9,10], salty seawater [11,12], saliva [13], and gasoline [14], or temperature along with moisture, termed hydrothermal aging [15,16,17], affects the properties of shapes obtained by a 3D printing method. The effects of prolonged immersion in water for as long as 248 days [18] and exposure to a solvent such as acetone [19] were also studied. A publication [6] also evaluated the effect of MO on PLA and PETG materials and their carbon fiber composites. The authors examined the effect of oil on materials with a sample fill of 30%, referring to data specified by the manufacturer for materials printed at 100%. The samples were exposed to MO for 7 days and 30 days at a constant temperature, and evaluated only for changes in tensile strength. The tests showed only an increase in Young’s modulus, and the conclusion was a decrease in the durability of the materials tested.

The automotive industry and other industries extensively use mineral oil as a lubricating and cooling medium in various machines and equipment. Often, additive technology prints come into contact with MO in various industrial applications, such as sorter components, prompting an understanding of how this interaction can affect the durability and strength of these components [19]. MO is one of the factors that can alter the properties of FDM prints, as it contains a complex mixture of chemicals that can interact with polymer plastics to varying degrees depending on temperature. Oil absorption, depending on the material and filling used, can affect the microstructure of the polymer material. It can migrate into the interior of the filament bundle and weaken the bonds between the polymer chains, leading to plasticization of the polymer and thus resulting in a significant change in the mechanical properties of these materials [20].

This article shows an experiment focused on analyzing the effects of MO on four different materials often used in 3D printing technology, i.e., ABS, ASA, PLA, and HIPS. The samples were placed under controlled conditions to observe the interaction with MO. Special attention was paid to changes in mechanical, structural, and chemical parameters. Juxtaposing the results before and after exposure to oil at two different temperatures made it possible to determine the effect of the simulated environment on the properties of 3D printed samples, adding to the knowledge in this area. This research extends the existing understanding of the changes that occur in the structure due to long-term exposure to MO, lasting up to 60 days. As an additional degradation factor, a storage temperature of 70 °C in MO was used. The research also adds to the understanding of changes in mechanical properties compared to reference samples not subjected to MO.

## 2. Materials and Methods

### 2.1. Materials and Printing Procedures

The filaments used for 3D printing were Smart ABS, HIPS-X, ASA 275, and PLA Premium, which are offered in Spectrum’s commercial distribution (Spectrum Company, Pęcice, Poland). All materials were polar white and had identical fiber diameters (1.75 mm) [21]. Details of the printing parameters depending on the type of material are shown in Table 1. Platinum classic mineral 15 W–40 mineral oil from Orlen S.A Płock (Poland) was used as the medium to simulate the environment in which the polymer fittings were placed. It is intended for use in automotive engines, both gasoline and diesel. According to the safety data sheet [22], this product contains in its composition, in addition to a mixture of liquid hydrocarbons of petrochemical origin, benzenesulfonic acid, methyl-mono-C20-24-branched alkyl derivatives, calcium salts (722503-68-6), calcium salts of alkyl (C18-C28) toluene sulfonic acid. 

A Zortrax M200 Plus printer (Zortrax S.A., Olsztyn, Poland) was used to print the samples. The dimensions and shape of the samples were made by standards for testing the mechanical properties of polymeric materials. The first standard PN-EN ISO 527-1 concerned the determination of mechanical properties in static tension of plastics [23], shapes of 150 × 20 × 4.

The second type of test sample was designed according to EN ISO 179-1 with dimensions of 80 × 10 × 4 without notch, which deals with Charpy impact determination [24].

### 2.2. Description of the Experiment Conducted

The study of the effect of the MO environment was conducted by completely immersing the shapes obtained from the 3D method at room temperature, i.e., 23 °C, and elevated temperature, i.e., 70 °C. The shapes shown were placed on metal trays with a ceramic layer so that the samples were not in contact with each other and were completely immersed in oil. Samples for testing were taken after 15, 30, and 60 days of exposure to oil. For comparative purposes, tests were also carried out on samples not exposed to MO. The samples were marked according to the scheme: ASA_15-T23 denotes a sample made of ASA exposed to oil at 15 days and 23 °C. To evaluate the structural changes, SEM observations were carried out, tensile mechanical properties were evaluated, and thermal stability changes were examined by TGA and material changes by FTIR.

### 2.3. Static Tensile Test

Tensile mechanical property tests were carried out on Type 1A fittings. Static tensile testing was carried out on a Zwick Roell Z010 testing machine (Zwick GmbH & Co., KG, Ulm, Germany). The tensile modulus (E_t_), tensile strength (σ_m_), elongation at maximum stress (ε_m_), and strain at break (ε_b_) were determined. The test parameters were as follows: pretension: 0.1 MPa; strain ratio in the elastic modulus determination area: 1 mm/min; strain ratio: 10 mm/min. At least 3 specimens were tested each time in each measuring system. Charpy impact tests were also carried out for standardized unnotched specimens according to [24] using a 4J hammer (Zwick GmbH & Co., KG, Ulm, Germany). At least 5 specimens in a series were tested each time. 

### 2.4. Morphological Analysis

Evaluation of structural changes was carried out for cryogenic breakthrough samples using a Jeol JSM-6480LV scanning electron microscope (SEM) (JEOL, Tokyo, Japan). Samples were sputter-coated with a platinum layer using a vacuum sputtering machine before testing. A cross-sectional scan of the reference samples was performed, as well as after the samples had been exposed to MO for 15 and 60 days, also at elevated temperatures. The anodic potential for the SEM was 1 kV, and images were taken at an approximation not exceeding 300×. 

### 2.5. Evaluation of Thermal Stability of Prints

To assess thermal stability, thermogravimetric (TGA) tests were carried out using a TG 209F3 apparatus from Netzsch Group (Selb, Germany). The measurement was carried out in the temperature range from 30 to 900 °C in a nitrogen atmosphere at a temperature rise rate of 10 °C/min. A sample (about 10 mg) was taken from the 3D printing samples. The thermal stability temperature was defined as the temperature at which a 5% loss in material weight was observed (T_5_). The temperatures at which 1%, 10%**,** and 50% mass loss in the sample occurred were also determined (T_1_, T_10_, T_50_). The temperature at which the most intense decomposition occurred was determined (T_DTG_) based on DTG curves. A description of the characteristic values is summarized in Table 2. Measurements were carried out with two repetitions for each material.

### 2.6. Fourier Transform Infrared Spectroscopy (FTIR)

Changes in the material were also studied by FTIR spectroscopy using Bruker’s Alpha instrument and ATR (reflection) technique from Bruker (Poznań, Poland). The measurement was carried out in the range from 4400 to 200 cm^−1^, and 32 scans with a resolution of 4 cm^−1^ were used.

### 2.7. Statistics

OriginPro 2024 Pro software with statistical analysis modules implemented was used to statistically analyze the results obtained. ANOVA with a post hoc Tukey test was used to compare the significantly different for each average value. The normal distribution was confirmed using the Shapiro–Wilk test, and Levene’s test confirmed the homogeneity of variance. All analyses were conducted assuming a significance level of *p* = 0.05.

## 3. Results

### 3.1. Tensile Test

The results obtained for all materials subjected to environmental conditions, i.e., oil over time and temperature, are presented and discussed in tabular form. The results will be presented separately for each material.

**Figure 1:**
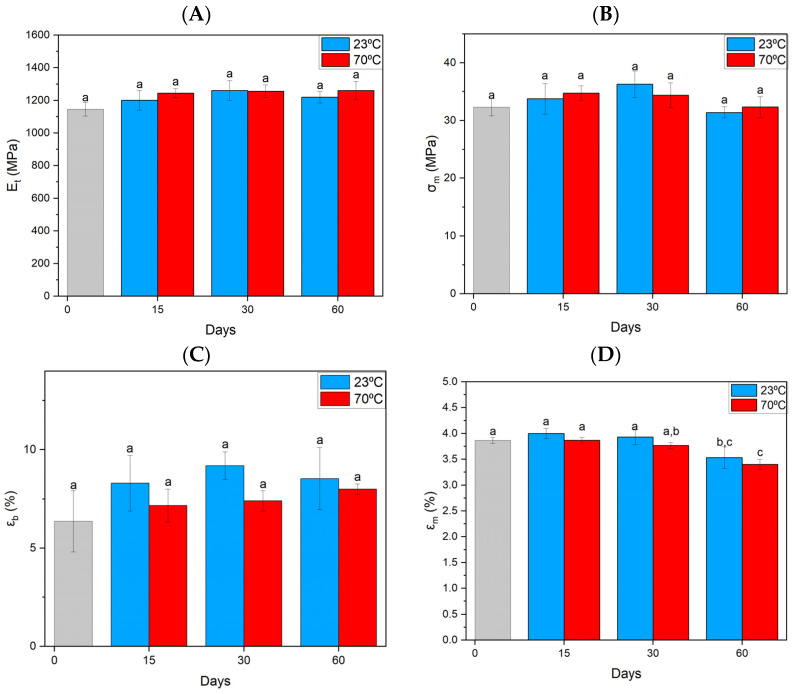
Summary of strength parameters for ABS material obtained from static tensile testing. (**A**) Elastic tangent modulus, (**B**) tensile strength, (**C**) strain at break, (**D**) elongation at maximum stress. Index indicates homogeneous groups within a single material. Letters (a,b,c) stand for homogeneous groups.

The ABS material showed no change in terms of decreases or increases in elastic tangential modulus—E_t_ (Figure 1A). The increase in tensile strength is most noticeable for the second time interval at room temperature only for a single sample—σ_m_ (Figure 1B). The strain at break—ε_b_ (Figure 1C) for individual samples not exposed to the additional factor of temperature showed a slight increase. The situation is different in the case of elongation at maximum stress—E_m_ (Figure 1D), as there is a noticeable decrease in the value with the samples exposed to MO for the longest time, which may be related to structural changes.

**Figure 2:**
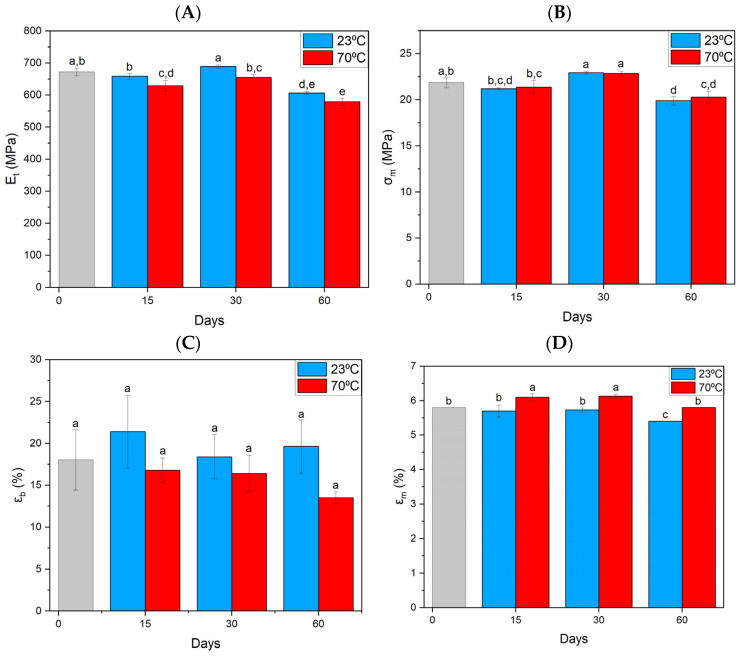
Summary of strength parameters for ASA material obtained from static tensile testing. (**A**) Elastic tangent modulus, (**B**) tensile strength, (**C**) strain at break, (**D**) elongation at maximum stress. Index indicates homogeneous groups within a single material. Letters (a,b,c,d,e) stand for homogeneous groups.

A tendency was found for the elastic modulus of the ASA material—E_t_ (Figure 2A) to decrease with the time of MO treatment. A significant change in tensile strength and strain at break does not accompany changes in elastic modulus. The observed changes in this parameter are small and within the standard deviation of the measurement. The strain at rupture—ε_b_ (Figure 2C) for specimens stored in MO is slightly higher, but only for the single specimens examined. We can observe the greatest changes for samples placed for 60 days in MO and a temperature of 23 °C, where the value of the parameter elongation at maximum stress decreases—ε_m_ (Figure 2D). For specimens exposed to elevated temperatures, a slight increase is seen after 15 and 30 days.

**Figure 3:**
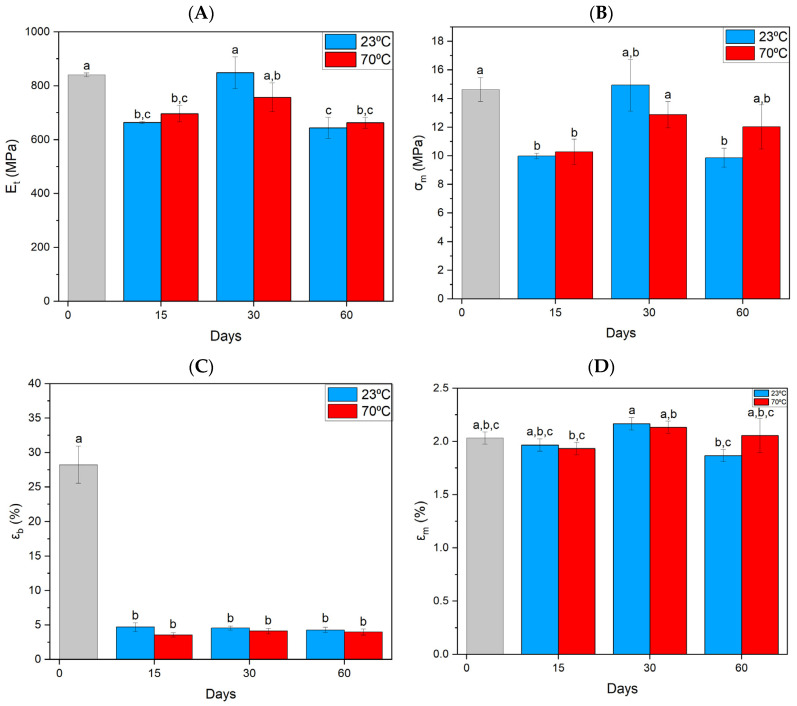
Summary of strength parameters for HIPS material obtained from static tensile testing. (**A**) Elastic tangent modulus, (**B**) tensile strength, (**C**) strain at break, (**D**) elongation at maximum stress. Index indicates homogeneous groups within a single material. Letters (a,b,c) stand for homogeneous groups.

The specimens made of HIPS material, as a result of MO, have decreased in almost all parameters determined by static tensile testing. Most noticeable is the decrease in the strain at break—ε_b_ (Figure 3C). Significant changes in the discussed parameters were found already after 15 days of aging. Further aging did not affect further deterioration of mechanical properties. It is worth noting the more than 50-fold reduction in strain at strength. 

**Figure 4:**
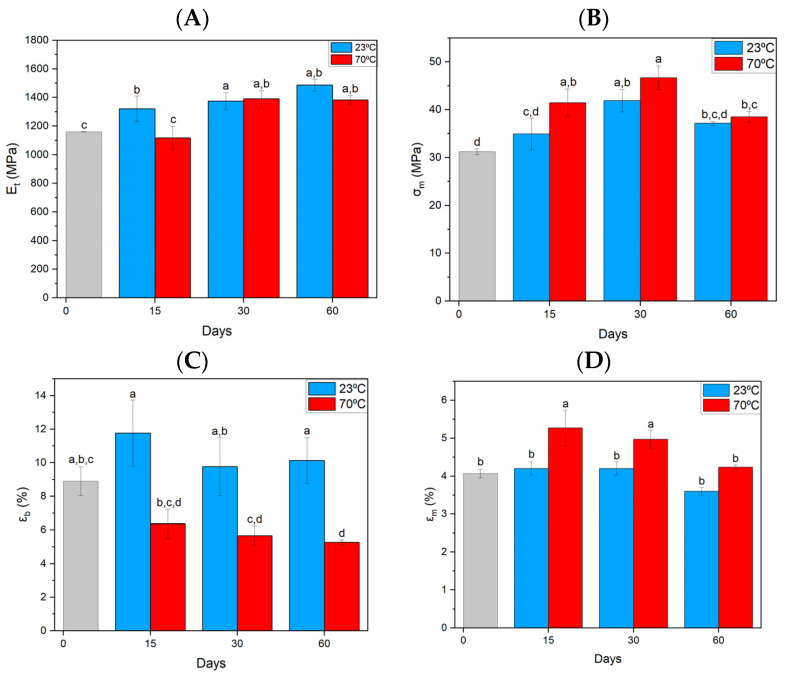
Summary of strength parameters for PLA material obtained from static tensile test. (**A**) Elastic tangent modulus, (**B**) tensile strength, (**C**) strain at break, (**D**) elongation at maximum stress. Index indicates homogeneous groups within a single material. Letters (a,b,c,d) stand for homogeneous groups.

PLA tends to increase in stiffness (-E_t_ Figure 4A) with aging time at both room and elevated temperatures. An increase in strength was also observed, with a decrease in this parameter after 60 days compared to 30 days, but the strength is still higher than the reference samples. Higher tensile strength is noticed more for samples stored at elevated temperatures—σ_m_ (Figure 4B). The observed relationships may be related to structural changes. The strain at break (Figure 4C—ε_b_) for the samples at the temperature stored at 23 °C is at the level of the reference ones. At an elevated temperature of 70 °C, a gradual significant decrease in the value of this parameter is noticeable. It is different in the case of elongation at maximum stress—ε_m_ (Figure 4D), which, in turn, the samples after 15 and 30 days show a slight increase relative to the reference and other samples.

### 3.2. Charpy Impact Test

**Figure 5:**
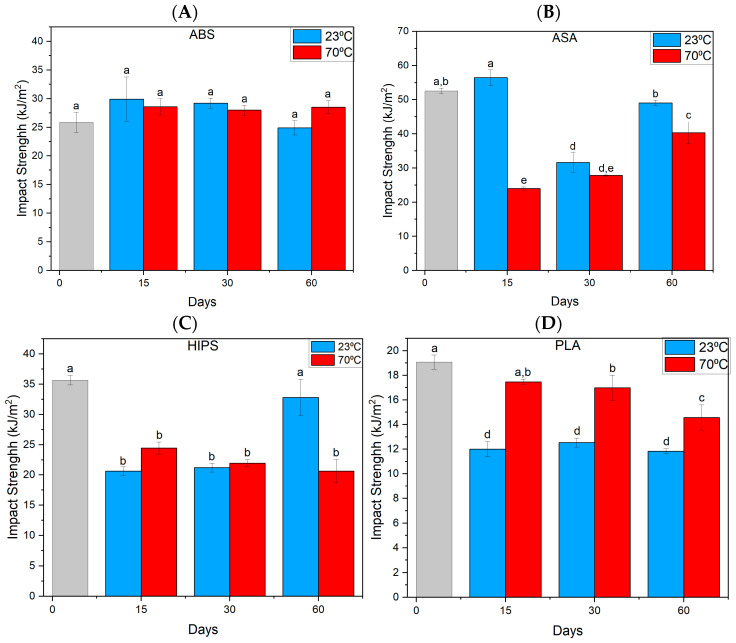
Summary of the Charpy impact strength test results of the samples. (**A**)—(ABS), (**B**)—(ASA), (**C**)—(HIPS), (**D**)—(PLA). Index indicates homogeneous groups within a single material. Letters (a,b,c,d,e) stand for homogeneous groups.

In the case of ABS material (Figure 5A), there were no significant changes in the impact strength values relative to the reference test. The situation is different for the ASA material (Figure 5B), where there is a noticeable decrease after 30 and 60 days of holding in MO. The largest decrease occurs for samples exposed to higher temperatures for 15 days, while for samples immersed in oil at room temperature, the results are at reference levels. In the case of the HIPS (Figure 5C) material, the decrease in the impact strength value already occurs after 15 days, and for samples exposed to higher temperatures, it persists regardless of the prolonged exposure to oil. Also, we can observe a noticeable effect of temperature and oil in the case of PLA material (Figure 5D), where after 15 days at 23 °C there is a decrease in impact strength and further aging does not affect further reduction of this parameter. On the other hand, at higher temperatures, a gradual decrease in impact strength was found successively after 30 and 60 days.

### 3.3. Scanning Electron Microscope

**Figure 6:**
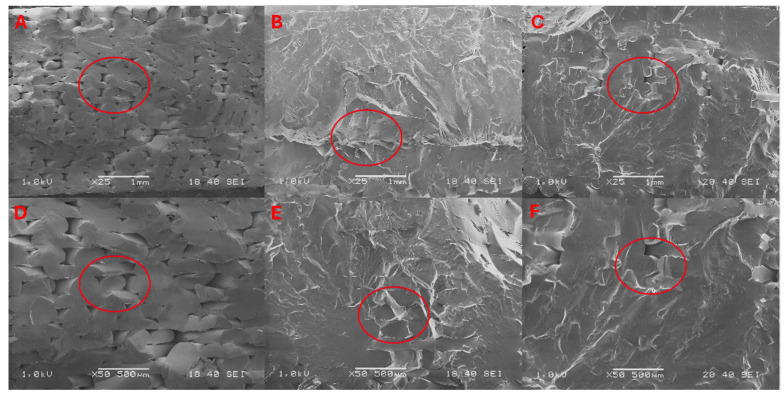
SEM images of the fracture surface of ABS reference samples (**A**,**D**) and images of the surface after exposure for 15 days (**B**,**E**) and 60 days (**C**,**F**) at a temperature of 23 °C.

**Figure 7:**
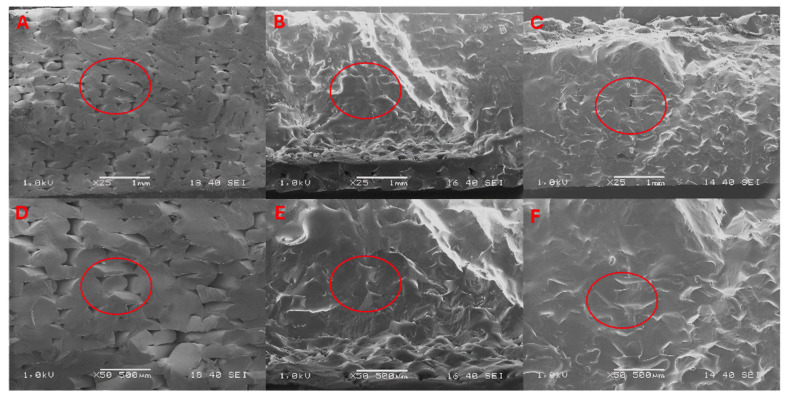
SEM images of the fracture surface of ABS reference samples (**A**,**D**) and images of the surface after exposure for 15 days (**B**,**E**) and 60 days (**C**,**F**) at a temperature of 70 °C.

The specimens were used for structural testing before mechanical tests, in which their cryogenic fracture surfaces were analyzed. The SEM images (Figure 6 and Figure 7) show images of the fracture surfaces of the aged samples and images of the surfaces of the samples after the 15 and 60 days in temperatures of 23 °C and 70 °C; the rest of the images are included in the supplements (Figures S1–S6). The red circles in the images represent the range being discussed.

The structure of the ABS material fittings (Figure 6) changed under MO exposure. The SEM image shows the fusion of individual filament paths already after 15 days of immersion in MO at post-cooling temperatures (B) and (E), which may indicate fusion of the material. On the other hand, delamination of the material is observed after 60 days (C) and (F), which may be due to prolonged exposure to MO. The structure shows changes including the blending of individual layers, which translates into mechanical properties, where an increase in stiffness due to MO exposure is noticeable. 

When MO is exposed to higher temperatures, changes in the structure of the ABS material due to degradation can be seen, which resulted in the merging of individual paths with changes in their alignment (B) and (E) after just 15 days. After 60 days of exposure to MO and higher temperatures, we can see an almost complete fusion of the individual gaps between layers (C) and (F), which is the reason for the increase in tensile strength and impact strength.

When analyzing the structure of the PLA material shaper (Figure S1), the SEM image shows a reduction in the gaps between individual bundles already after 15 days of MO treatment (B) and (E); the height of the layer stacking is almost as on the reference sample. On the shapes treated with oil at 60 days, the complete disappearance of gaps between individual filament bundles is noticeable, and the melting of adjacent bundles is also visible (F). This effect may be the reason for the highest strength of these materials among the samples tested in earlier time intervals.

As in the case of MO exposure at room temperature to PLA shapes, an even greater reduction in the space between individual layers is noticeable after just 15 days (E). On the other hand, after prolonged exposure to MO at a higher temperature (Figure S2), a smoothing of the middle layer in the sample is noticeable, which negatively translated into tensile strength, but positively the temperature factor affected impact strength. 

In the case of fittings made of ASA material (Figure S3), we can already see from the structure of the reference fittings that the alignment of individual layers is not preserved correctly, and the reference fittings of PLA material (Figure S2) can serve as an example. After 15 days of exposure to MO, the bonding of individual bundles of filament layers (E) is noticeable. After reaching the maximum holding time in the oil, even more bonding of individual layers is visible (F), in addition, gaps between the bundles are preserved in the middle layers, which may be related to the lack of migration to the center of the oil. The strength decreased, but there was a noticeable increase in strain at break for the samples stored the longest.

The structure of ASA samples that were stored oil at a higher temperature (Figure S4) shows more uneven bonding of the center fibers (E). The higher temperature may have caused even more visible overlapping and smoothing of adjacent layers and microcracks (F), which translated into even lower strength.

Shapes made of HIPS material (Figure S5) already show significant changes in structure related to the arrangement of the filament fibers after 15 days. There is a noticeable merging of individual layers and their smoothing (E), which is even more noticeable for samples exposed for 60 days (F). Gap behavior is visible throughout the sample, which may indicate oil migration into the inner layers of the shaper. This may have had a positive effect on increasing the strength of the samples after 15 days, where the changes not in fiber alignment were not so great.

HIPS material samples treated with MO at a higher temperature (Figure S6) show the vertical fusion of individual layers, thus leaving room for oil migration (E). Samples kept in oil for 60 days are characterized by a significantly more visible unification of the filament fiber structure and its melting (F).

### 3.4. Analysis of TGA Results

**Figure 8:**
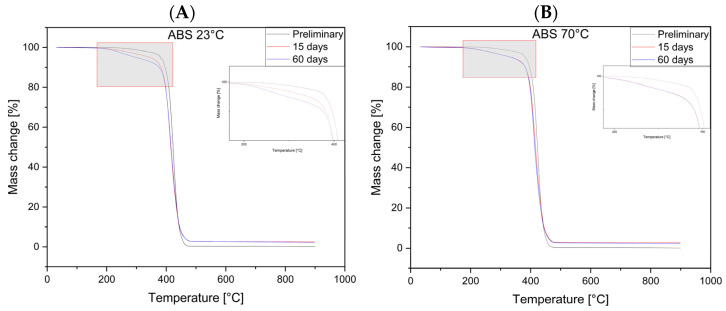
TGA plots of samples of ABS without treatment and after 15 and 60 days. (**A**)—Temperature 23 °C, (**B**)—Temperature 70 °C.

Figure 8 shows TGA thermograms of ABS samples from the test materials treated in MO for 15 and 60 days at 23 °C and 70 °C and the virgin material. The TGA waveforms of polymer samples treated with MO regardless of time and temperature are similar to each other. Decomposition occurs similarly to that of the virgin material, but differences are evident in the initial stage of decomposition of the polymer materials.

#### 3.4.1. Mineral Oil

Decomposition of machine MO occurs in the range of about 230–450 °C (Figure S7), which is consistent with [25,26], and the mass loss in this range was 94%. In the range of 200–350 °C, there is both evaporation of low molecular weight hydrocarbons and oil degradation, and above 350 °C degradation of long-chain hydrocarbons [27]. An analysis of the data in Table 2 shows that this oil has lower thermal stability than the polymeric materials tested; the onset of its degradation defined as T_5_ is about 241 °C.

Analysis of the data in Table 2 shows that this oil has lower thermal stability than the polymer materials tested; the onset of its decomposition defined as T_5_ is about 241 °C.

#### 3.4.2. PLA

In the case of PLA, the highest thermal stability was observed in the shapes not treated with MO. Immersion in MO reduced the thermal stability of PLA material (Figure S8), especially when the oil was operated at elevated temperatures. The sample kept in oil for 60 days at 70 °C had the lowest thermal stability temperature. From the course of TGA curves, it can be concluded that the simultaneous action of oil and elevated temperature for an extended period causes significant changes in the thermal stability of PLA shapes and thus can affect the change of other properties, especially mechanical properties. The T_5_ value of PLA samples held at 23 °C decreased from about 330 °C for unmodified PLA to 310 °C for PLA held for 60 days in MO. Significantly greater differences were noted for samples exposed to oil at 70 °C; here, a reduction in the T_5_ value of the sample after 60 days to 283 °C was noted. T_50_ values were similar and ranged from 356 to 353 °C.

#### 3.4.3. HIPS

Similar behavior was also observed for samples made of HIPS. As the MO time increased, the material showed lower thermal stability (Figure S9) and the increased storage temperature caused a more pronounced decrease in the thermal stability of the material. The T_5_ value decreased from 380 °C to 349 °C when the materials were stored at 23 °C and to 293 °C when the samples were stored in MO at 70 °C 

#### 3.4.4. ASA

The ASA material also showed a similar trend of reduced thermal stability (Figure S10). Larger differences in the reduction of T_5_ were observed when the ASA material was exposed to oil for 30 days at elevated temperatures. On the other hand, after 60 days, the value of T_5_, regardless of temperature, coincided and was 287 °C, which may indicate that after this immersion in MO, temperature has a lower effect on degradation than oil time.

#### 3.4.5. ABS

The observed changes in the values of the weight loss temperature of the sample in the initial stages of decomposition (T_1_ and T_5_) may be related to the presence of a MO layer on the structures of the filament (Figure S11). From the TGA testing of the MO sample, we determined its onset of decomposition temperature T_5_, which occurs at lower values than for the polymer materials used. Taking into account that a layer of MO remains on the surface of the samples and due to the porous nature of the structure of the samples obtained by the 3D method, this oil could penetrate the center of the sample, and the observed effects of lower values of weight loss temperature could be related to the presence of MO with lower thermal stability. However, an additional effect of increased temperature is observed (Figure S12). The lower values of T_1_ and T_5_ along with the increase in the time the oil acts on the sample material at elevated temperatures confirm the degrading effects of the conditions on PLA, ASA, and HIPS material in particular. The degradation effects would need to be confirmed by FTIR analysis.

In addition, MO migrating into the sample structure could penetrate between the polymer macromolecules, causing a change in their intramolecular interactions and plasticization of the material. However, these suppositions were not confirmed by mechanical tests, in which no significant changes in the elongation of the sample or reduction in the elastic modulus were observed. Therefore, it can be concluded that a layer of oil is formed on the surface in the filament fibers of the porous structure of the sample, which is consistent with a report [27] where PLA was used as a superhydrophobic polylactic aerogel capable of recovering oil spills.

Decomposition of machine-made MO occurs in the range of about 230–450 °C, which is consistent with [25,26], with a mass loss of 94%. In the range of 200–350 °C, both evaporation of low molecular weight hydrocarbons and oil degradation occur, and above 350 °C, the degradation of long-chain hydrocarbons occurs [27].

An analysis of the data in the table shows that the oil has lower thermal stability than the polymer materials tested; the onset of its degradation defined as T_5_ is about 241 °C.

#### 3.4.6. PLA

In the case of PLA, the highest thermal stability was observed in the shapes not treated with MO. Immersion in MO reduced the thermal stability of PLA material, especially when the oil was operated at elevated temperatures. The sample kept in oil for 60 days at 70 °C had the lowest thermal stability temperature (Figure S13). Based on the TGA curves, it can be concluded that the simultaneous action of oil and elevated temperature for an extended period causes significant changes in the thermal stability of the PLA shapes and thus can change other properties, especially mechanical properties.

#### 3.4.7. HIPS

Similar behavior was also observed for samples made of HIPS (Figure S14). With increasing oil exposure time, the material exhibited lower thermal stability, and increased storage temperature resulted in a more pronounced decrease in the thermal stability of the material.

#### 3.4.8. ASA

The ASA material also showed a similar trend (Figure S15) of decreased thermal stability. The observed changes in the values of the sample’s weight loss temperature at the onset of decomposition (T_1_ and T_5_) may be related to the presence of a MO layer on the filament structures and its partial migration into the filament material. From the TGA testing of the engine oil sample, its onset of decomposition temperature was determined, as the value of T_1_ and T_5_. These values are lower than in the case of the materials used, which occurs in the interval 96.6 and 236.6, i.e., lower than the polymer materials used. Given that a layer of MO remains on the surface of the samples, and due to the porous structure of the samples obtained by the 3D method, this oil could penetrate the center of the sample, and the observed effects of lower values of mass loss temperature could be related to the presence of MO. The lower values of T_1_ and T_5_ along with the increase of the immersion time and at elevated temperatures may also confirm the increased penetration of the oil into the interior of the sample. The degradation effects would need to be confirmed by FTIR analysis.

In addition, MO migrating into the structure of the sample could penetrate between the polymer macromolecules, causing a change in their intramolecular interactions and plasticizing the material. However, these suppositions were not confirmed by mechanical tests, in which no significant changes in the elongation of the sample or reduction in the elastic modulus were observed. Therefore, it can be concluded that a layer of oil is formed on the surface in the filament fibers of the porous structure of the sample, which is consistent with a report [26] where PLA was used as a superhydrophobic polylactic aerogel capable of recovering oil spills.

### 3.5. Analysis of FTIR Results

#### 3.5.1. Mineral Oil 

The spectrum of the mineral oil used is shown in Figure S16. In the course of the spectrum, characteristic bands were observed at 2952 cm^−1^, 2920 cm^−1^, and 2850 cm^−1^ corresponding to -CH2-CH3 groups, as well as a band of 1720 cm^−1^ (C=O), 1500 cm^−1^ and 1376 cm^−1^ corresponding to -CH2 and 1050 cm^−1^ (C-O), 722 cm^−1^ (-CH2). As a base, the MO contains a mixture of hydrocarbons of different chain lengths, which is confirmed by FTIR results indicating the presence of -CH2 and -CH3 groups. The presence of these bands observed for MO coincides with the bands seen for the polymers used. In addition, a residual MO layer was also observed on the filament fibers, which makes it difficult to analyze the FTIR results of the polymer materials.

MO also contains many functional additives such as corrosion inhibitors, oxidation inhibitors, detergents, viscosity modifiers, and many multifunctional additives [28,29].

**Figure 9:**
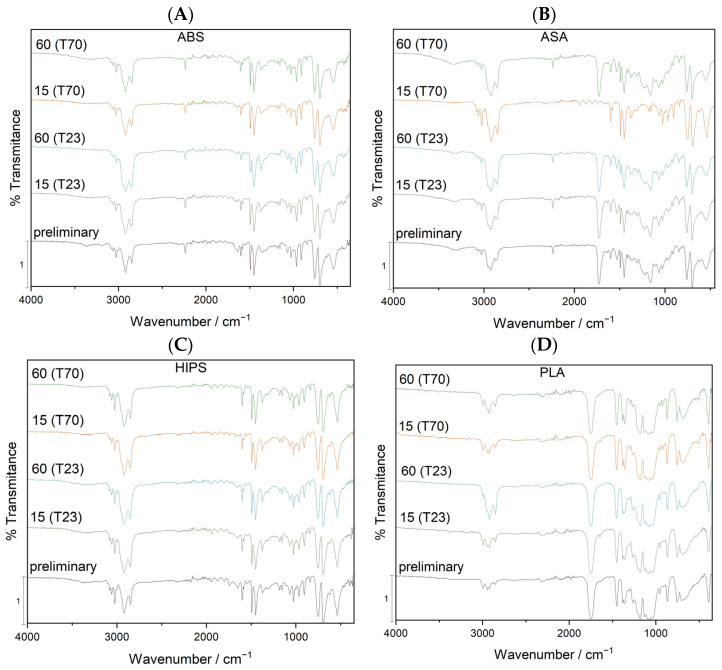
FTIR absorption spectra for ABS (**A**), ASA (**B**), HIPS (**C**), and PLA (**D**).

Figure 9 shows selected FTIR spectra of the original sample after 60 days of oil exposure at 23 °C and 70 °C. Regardless of the oil exposure time and temperature, the course of FTIR spectra is typical for the polymers studied.

In the case of PLA, slight changes were observed in the increase in the intensity of wavelengths 1580, 1480, and 1280, which may indicate the presence of residual MO. A decrease in the peak of 1263 cm^−1^ and an increase to 1293 cm^−1^ were observed, which suggests the degradation of ketone groups and the appearance of vinyl groups [30,31]. 

Only on the PLA 60 T70 spectrum, an increase in peak intensity was observed at a wavelength of about 922 cm^−1^, indicating the presence of α crystals [32], which may indicate the degradation processes taking place changing the matrix structure. Also, the observed intensity changes in the range of 685–760 cm^−1^ can be associated with crystallinity changes that lead to other conformations of the polymer [33]. The observed changes in the course of the spectra may be due to the initiation of degradation of PLA macromolecules, especially in the extended period of oil at elevated temperature [33]. However, the observed changes are small. 

#### 3.5.2. HIPS

In the course of the FTIR spectrum for the sample after 60 days, especially at elevated temperature, slight changes were observed in the carbonyl region (>C=O) (1800–1620 cm^−1^), especially at 1730 and 1648 cm^−1^, associated with the formation of saturated ketones, unsaturated ketones, aldehydes, and others as products of the initial degradation processes [34]. These changes were more pronounced for the sample held at elevated temperatures.

#### 3.5.3. ASA

FTIR spectra of ASA samples subjected to long-term MO treatment show some changes in the course that may indicate structural changes in the material. The appearance of a broad peak in the 3000–2700 cm^−1^ range associated with the presence of OH groups was observed. The observed changes in the 1400 cm^−1^ to 1200 cm^−1^ range could be related to ester formation occurring during degradation [35]. The 60T70 sample as well as the 60T23 sample show structural changes associated with oil exposure, which could affect the performance properties studied.

#### 3.5.4. ABS

The ABS spectra obtained are similar regardless of the MO time and temperature. The spectrum obtained for the 60 T70 sample shows some changes in the waveform compared to the unmodified or 60 T23. These changes caused by additional heat exposure may indicate slight structural changes in the material. Minor changes were observed from 3000 to 3700 cm^−1^ associated with -OH stretching. Nonsignificant differentials also occur in the 1200–800 cm^−1^ area associated with C-O bonds and in the 1600–1800 cm^−1^ area associated with C=0, indicating nonsignificant oxidegradation changes, especially in the case of the 60 T70 sample [35].

## 4. Conclusions

PLA, ABS, HIPS, and ASA are widely used in various fields of technology. Mineral oil, due to its widespread use for lubricating moving machine parts, is very common in the working environment of machines including parts made in 3D printing. After evaluating the effect of mineral oil on the properties of the materials studied, it can be stated that its presence had different effects on the materials studied. 

In our opinion, ABS is the most suitable of the studied materials for working in a mineral oil environment. It is the least sensitive to the presence of oil; its mechanical properties under MO are the most stable and do not change much even at elevated temperatures. Changes in the structure of ABS were also far less significant compared to the other plastics examined. However, further research is needed to confirm the stability of the material’s shape and dimensions under such operating conditions. With very complex shapes and large part dimensions, deformations can occur that prevent correct work. This needs to be verified, so further work by the authors will be carried out in this direction.

In the case of other plastics, the stability of mechanical parameters is not as good. The general trend for the other materials is a decrease in impact strength (related to the impact strength value measured in the reference group for each material). For parts made of these materials, this will result in lower impact resistance, with a consequent higher susceptibility to damage and fracture during the working of parts.

## Figures and Tables

**Table 1:** 3D printing parameters.

Parameter Name	Smart ABS	HIPS-X	ASA 275	PLA Premium
Product Symbol	5903175658173	5903175658012	5903175653086	5903175657114
Extrusion Temperature	275 °C	250 °C	240 °C	210 °C
Platform Temperature	80 °C	80 °C	60 °C	30 °C
Density	1.05 g/cm^3^	1.05 g/cm^3^	1.08 g/cm^3^	1.24 g/cm^3^
Infill pattern	Rectilinear			
Fill angle	45°			
Infill density	100%			
Fill density at the bottom	100%			
Fill density on the wall	100%			
Layer height	0.3 mm			
Solid layers	Top 7, Bottom 4			
Print speed	30 mm/s			
Nozzle diameter	0.4 mm			
Diameter tolerance	±0.03			
Layer height	0.19 mm			
Filling density	100%			

**Table 2:** The TGA analysis results.

Material			T_1_	T_5_	T_50_	T_onset_	T_DTG_
	Temperature (°C)	Time (days)	(°C)	(°C)	(°C)	(°C)	(°C)
Mineral oil	-	-	141.6 (0.1)	241.5 (0.1)	323.6 (0.3)	275.0 (0.2)	331.0 (0.1)
PLA	-	0	306.2 (0.3)	329.8 (0.5)	356.2 (0.1)	341.3 (0.3)	360.2 (0.5)
	23	15	214.3 (0.5)	319.2 (0.2)	355.0 (0.2)	338.3 (0.1)	360.4 (0.2)
		30	212.7 (0.1)	315.0 (0.6)	354.0 (0.3)	338.7 (0.2)	358.8 (0.1)
		60	211.2 (0.4)	310.8 (0.1)	353.0 (0.3)	339.1 (0.1)	357.3 (0.4)
	70	15	214.5 (0.2)	313.8 (0.1)	355.4 (0.3)	338.5 (0.1)	359.5 (0.2)
		30	207.4 (0.4)	301.5 (0.2)	354.2 (0.1)	344.1 (0.2)	357.4 (0.3)
		60	200.3 (0.3)	289.3 (0.1)	353.0 (0.5)	349.8 (0.3)	355.4 (0.4)
HIPS	-	0	288.9 (0.1)	380.4 (0.2)	422.9 (0.1)	408.6 (0.3)	425.7 (0.1)
	23	15	244.1 (0.3)	349.4 (0.1)	422.3 (0.2)	401.8 (0.3)	424.7 (0.1)
		30	239.8 (0.4)	349.4 (0.5)	422.1 (0.6)	402.4 (0.2)	425.3 (0.4)
		60	235.5 (0.2)	349.4 (0.1)	421.9 (0.3)	403.0 (0.4)	426.0 (0.2)
	70	15	241.5 (0.2)	333.3 (0.1)	421.8 (0.5)	400.0 (0.3)	425.2 (0.2)
		30	224.5 (0.1)	313.5 (0.3)	421.8 (0.2)	403.2 (0.4)	425.4 (0.2)
		60	207.5 (0.4)	293.8 (0.2)	421.9 (0.4)	406.5 (0.3)	425.6 (0.1)
ASA	-	0	270.6 (0.2)	322.5 (0.3)	407.1 (0.1)	391.2 (0.4)	406.6 (0.1)
	23	15	221.6 (0.1)	296.9 (0.5)	406.9 (0.2)	382.4 (0.4)	405.0 (0)
		30	221.6 (0.3)	291.9 (0.3)	405.9 (0.2)	386.2 (0.1)	405.0 (0)
		60	221.6 (0.4)	287.0 (0.4)	405.0 (0.2)	390.1 (0.3)	405.0 (0)
	70	15	204.7 (0.2)	277.7 (0.3)	403.3 (0.1)	380.2 (0.5)	404.8 (0.2)
		30	213.1 (0.1)	282.3 (0.6)	404.1 (0.2)	385.1 (0.3)	404.9 (0.1)
		60	221.6 (0.4)	287.0 (0.4)	405.0 (0.1)	390.1 (0.1)	405.0 (0)
ABS	-	0	219.8 (0.1)	344.6 (0.1)	415.2 (0.3)	387.0 (0)	412.3 (0.1)
	23	15	187.0 (0.3)	267.9 (0.1)	414.7 (0.2)	391.1 (0.2)	411.4 (0.1)
		30	196.1 (0.2)	282.9 (0.3)	415.2 (0.1)	390.8 (0.1)	412.8 (0.2)
		60	205.3 (0.4)	298.0 (0.4)	415.8 (0.1)	390.6 (0.2)	414.3 (0)
	70	15	212.9 (0.1)	328.4 (0.5)	416.3 (0.1)	390.0 (0.1)	414.8 (0.1)
		30	211.0 (0.4)	328.7 (0.3)	415.0 (0.6)	390.1 (0.2)	412.2 (0.3)
		60	209.1 (0.3)	329.0 (0.2)	413.8 (0.4)	390.3 (0.1)	409.7 (0.1)

## Data Availability

The raw data supporting the conclusions of this article will be made available by the authors on request.

## Supplementary Materials

The following supporting information can be downloaded at: https://www.mdpi.com/article/10.3390/ma17153680/s1, Figure S1: SEM images of the fracture surface of PLA reference samples (A) and (D) and images of the surface after exposure for 15 days (B) and (E) and 60 days (C) and (F) At temperature 23 °C. Figure S2: SEM images of the fracture surface of PLA reference samples (A) and (D) and images of the surface after exposure for 15 days (B) and (E) and 60 days (C) and (F) At a temperature of 70 °C. Figure S3: SEM images of the fracture surface of ASA reference samples (A) and (D) and images of the surface after exposure for 15 days (B) and (E) and 60 days (C) and (F) In temperatures 23 °C. Figure S4: SEM images of the fracture surface of ASA reference samples (A) and (D) and images of the surface after exposure for 15 days (B) and (E) and 60 days (C) and (F) At a temperature of 70 °C. Figure S5: SEM images of the fracture surface of HIPS reference samples (A) and (D) and images of the surface after exposure for 15 days (B) and (E) and 60 days (C) and (F) In temperatures 23 °C. Figure S6: SEM images of the fracture surface of HIPS reference samples (A) and (D) and images of the surface after exposure for 15 days (B) and (E) and 60 days (C) and (F) In temperature 70 °C. Figure S7: TGA plots of mineral oil. Figure S8: TGA plots of PLA samples without treatment and after 15 and 60 days at 23 °C. Figure S9. TGA plots of HIPS samples without treatment and after 15 and 60 days at 23 °C. Figure S10: TGA plots of ASA samples without treatment and after 15 and 60 days at 23 °C. Figure S11: TGA plots of ABS samples without treatment and after 15 and 60 days at 23 °C. Figure S12: TGA plots of ABS samples without treatment and after 15 and 60 days at 70 °C. Figure S13: TGA plots of PLA samples without treatment and after 15 and 60 days at 70 °C. Figure S14: TGA plots of HIPS samples without treatment and after 15 and 60 days at 70 °C. Figure S15: TGA plots of ASA samples without treatment and after 15 and 60 days at 70 °C. Figure S16: FTIR absorption spectra for mineral oil.
