# Supplementary figures and images for: Evaluation of the Effect of Mineral Oil Exposure on Changes in the Structure and Mechanical Properties of Polymer Parts Produced by Additive Manufacturing Techniques
Source: Materials (Basel). 2024 Jul 25;17(15):3680. doi: 10.3390/ma17153680 (PMC11313062; doi:10.3390/ma17153680)

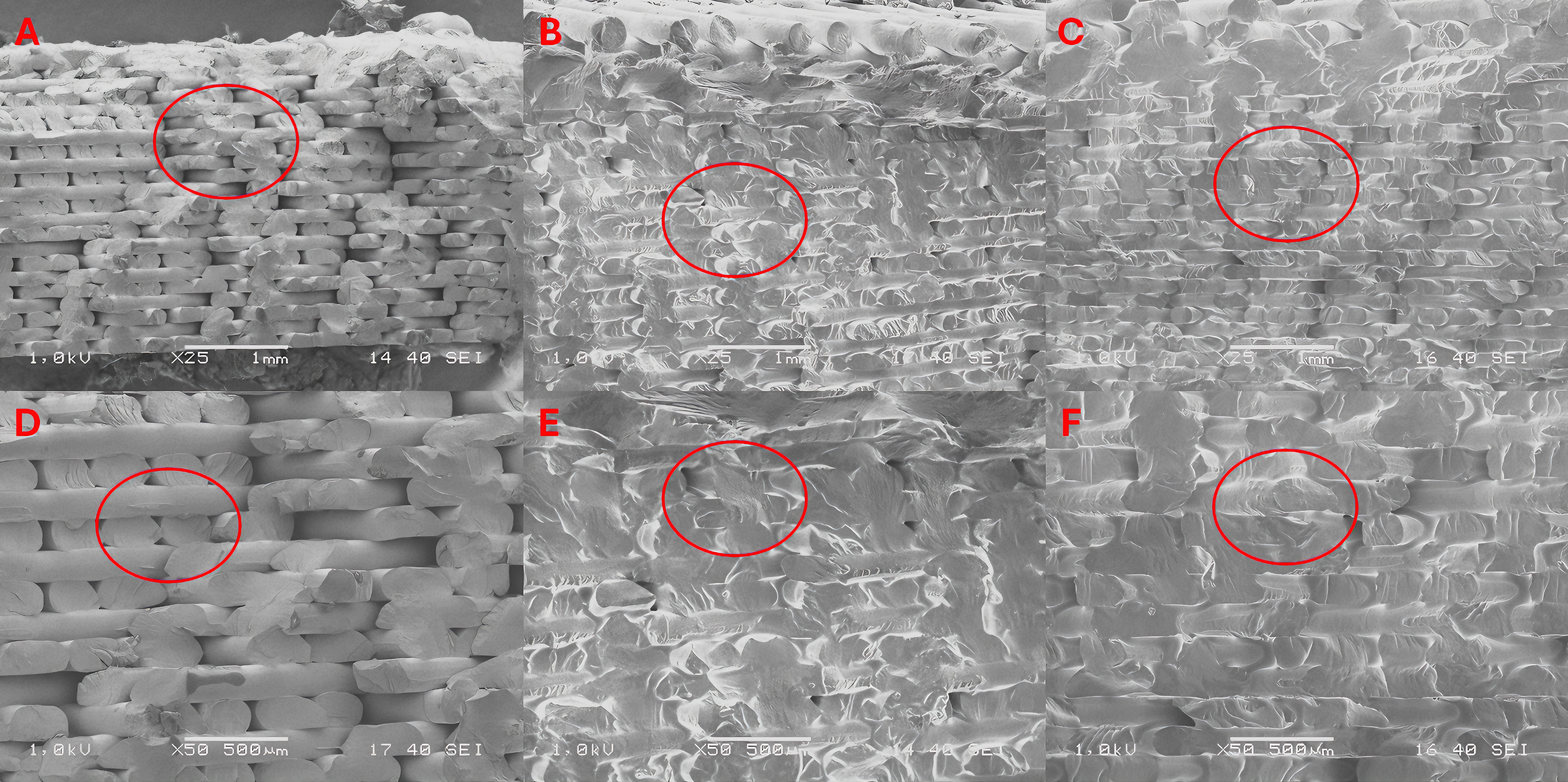

Supplement: Supplementary file 1 [file materials-17-03680-s001.zip › Figure S1.png]

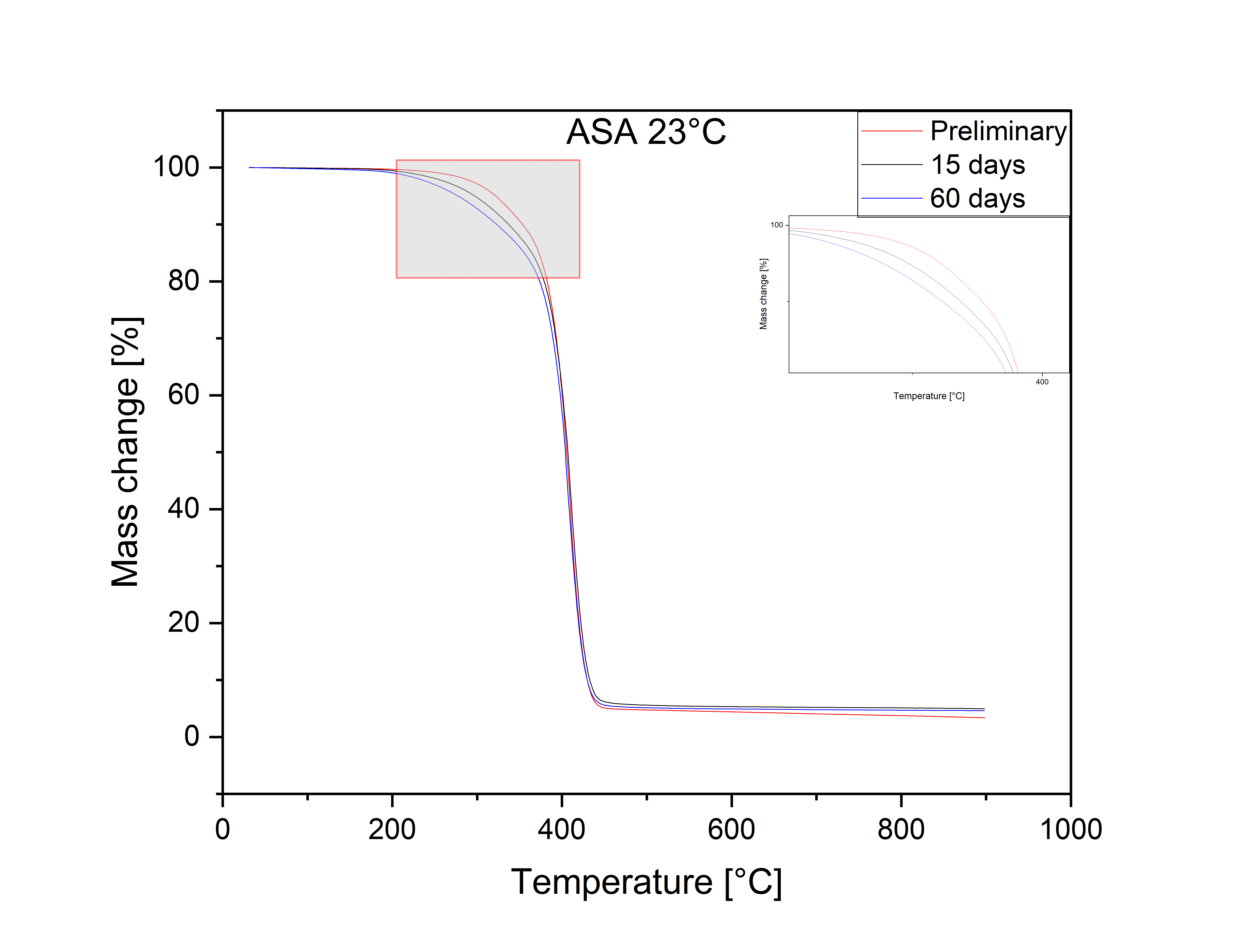

Supplement: Supplementary file 1 [file materials-17-03680-s001.zip › Figure S10.png]

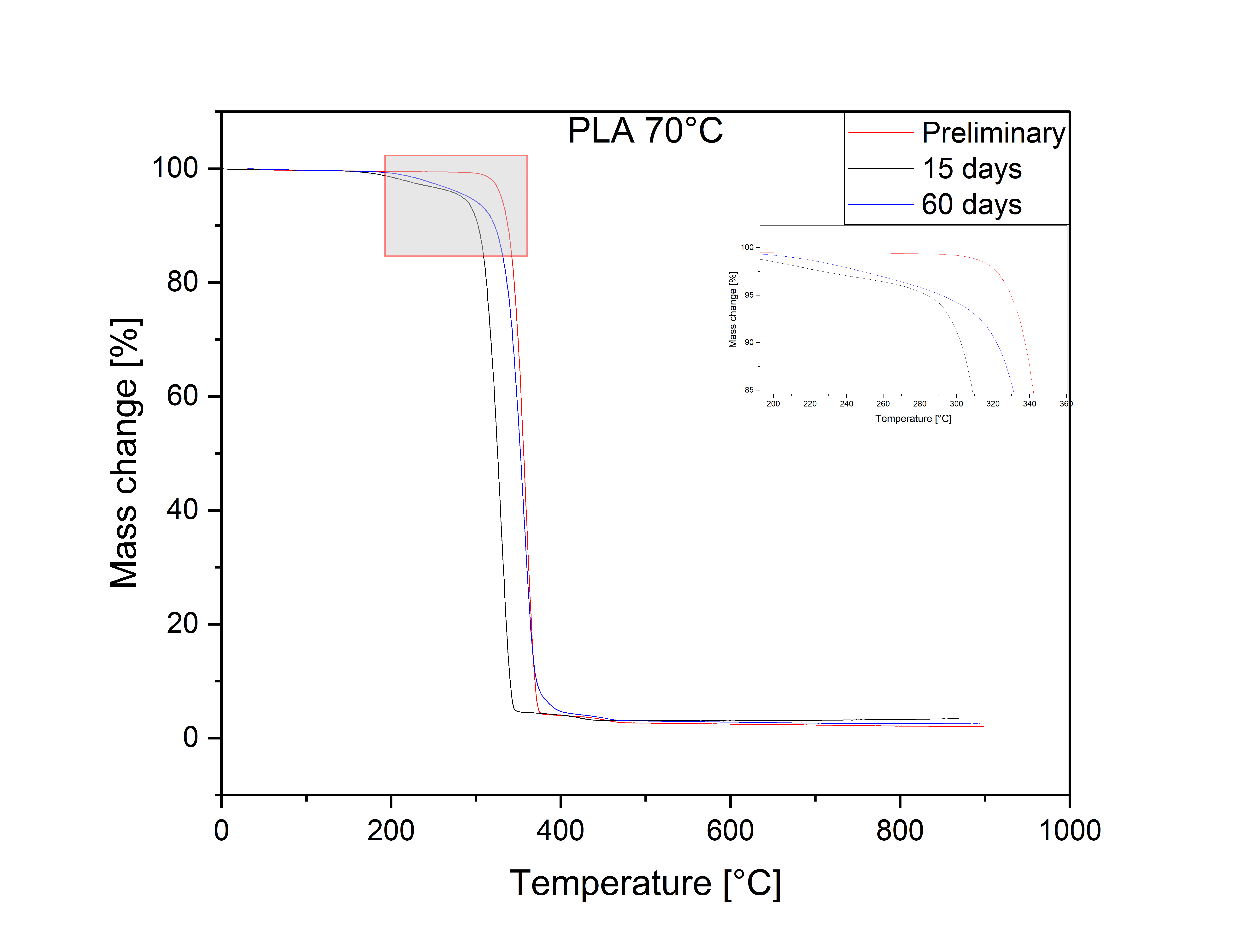

Supplement: Supplementary file 1 [file materials-17-03680-s001.zip › Figure S13.png]

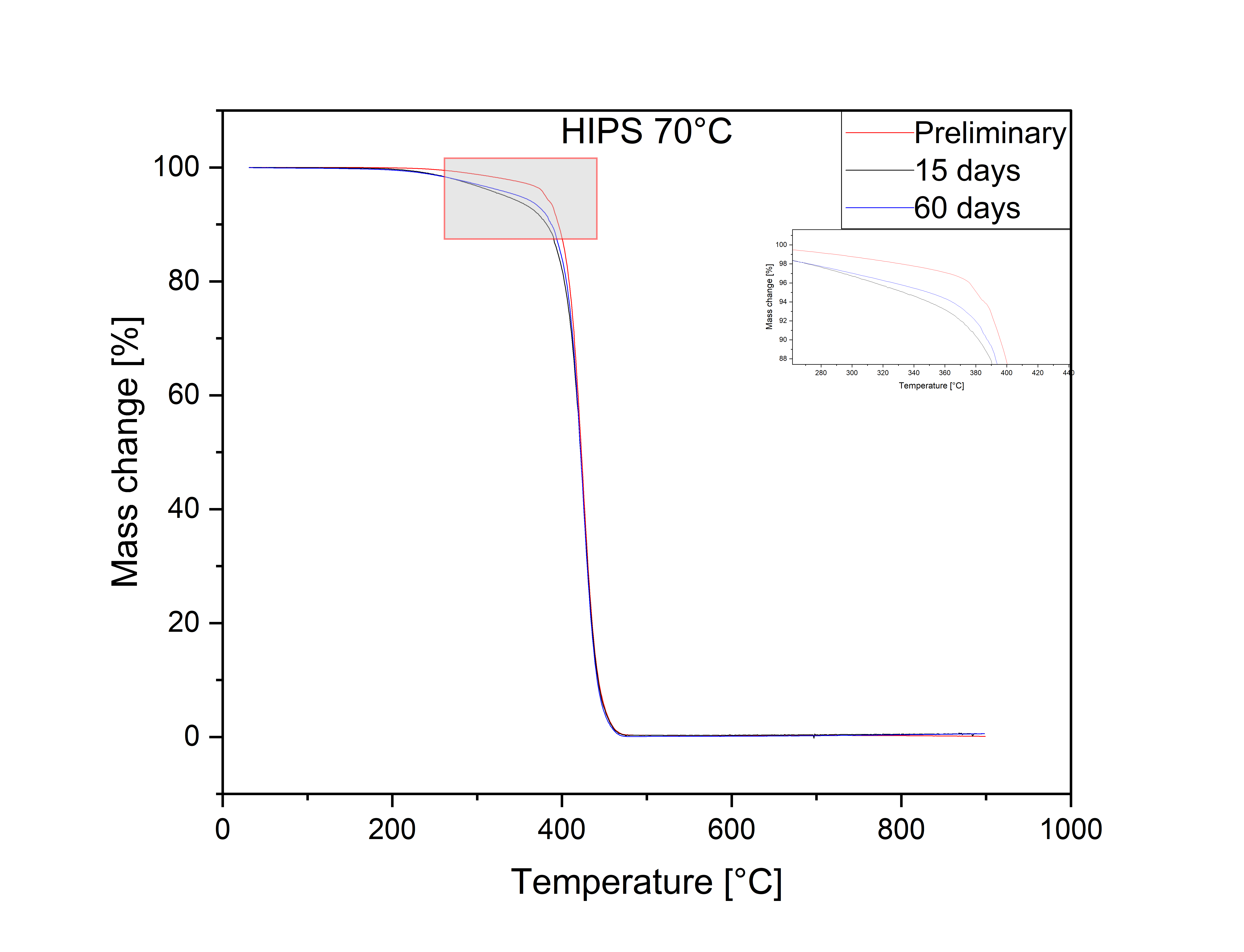

Supplement: Supplementary file 1 [file materials-17-03680-s001.zip › Figure S14.png]

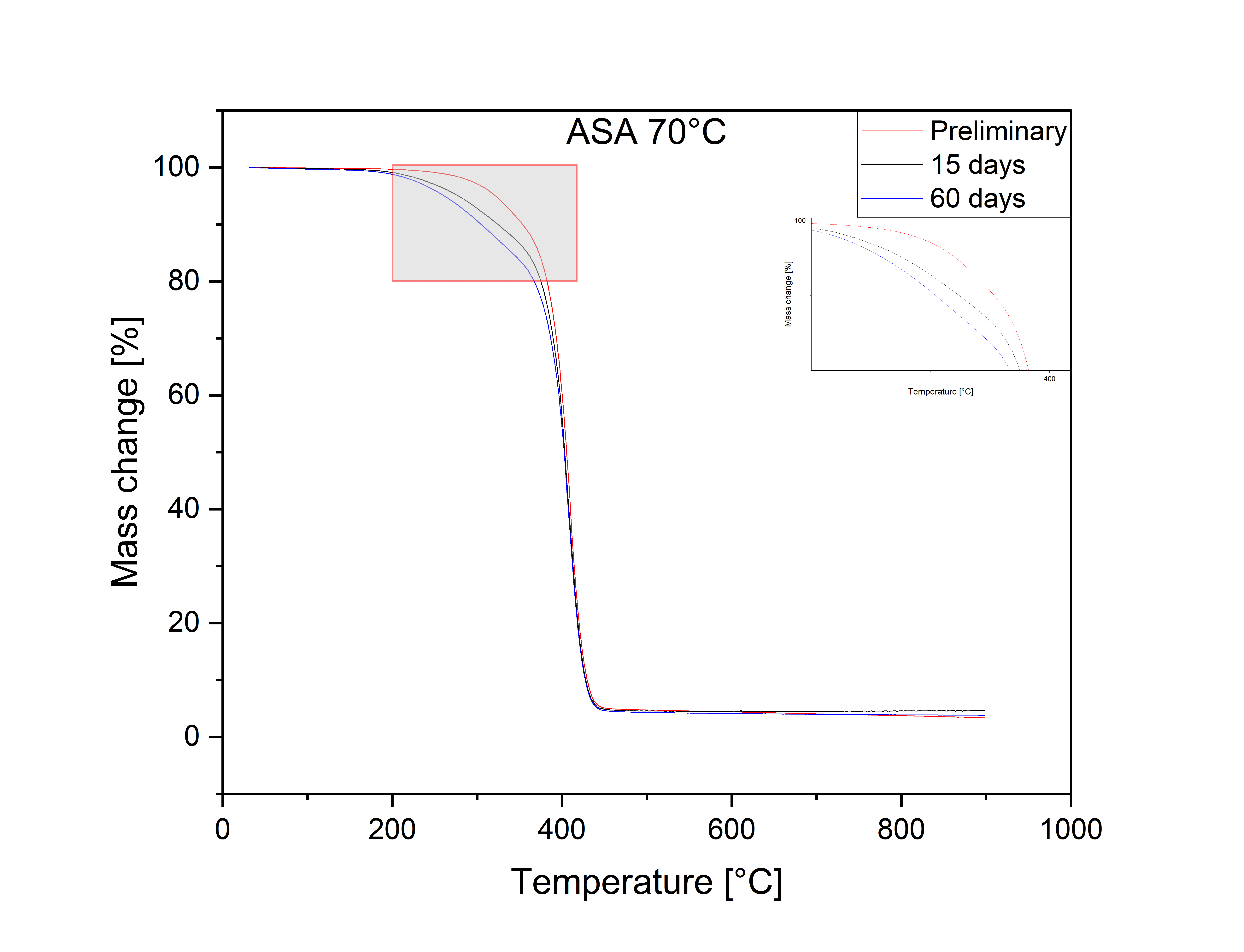

Supplement: Supplementary file 1 [file materials-17-03680-s001.zip › Figure S15.png]

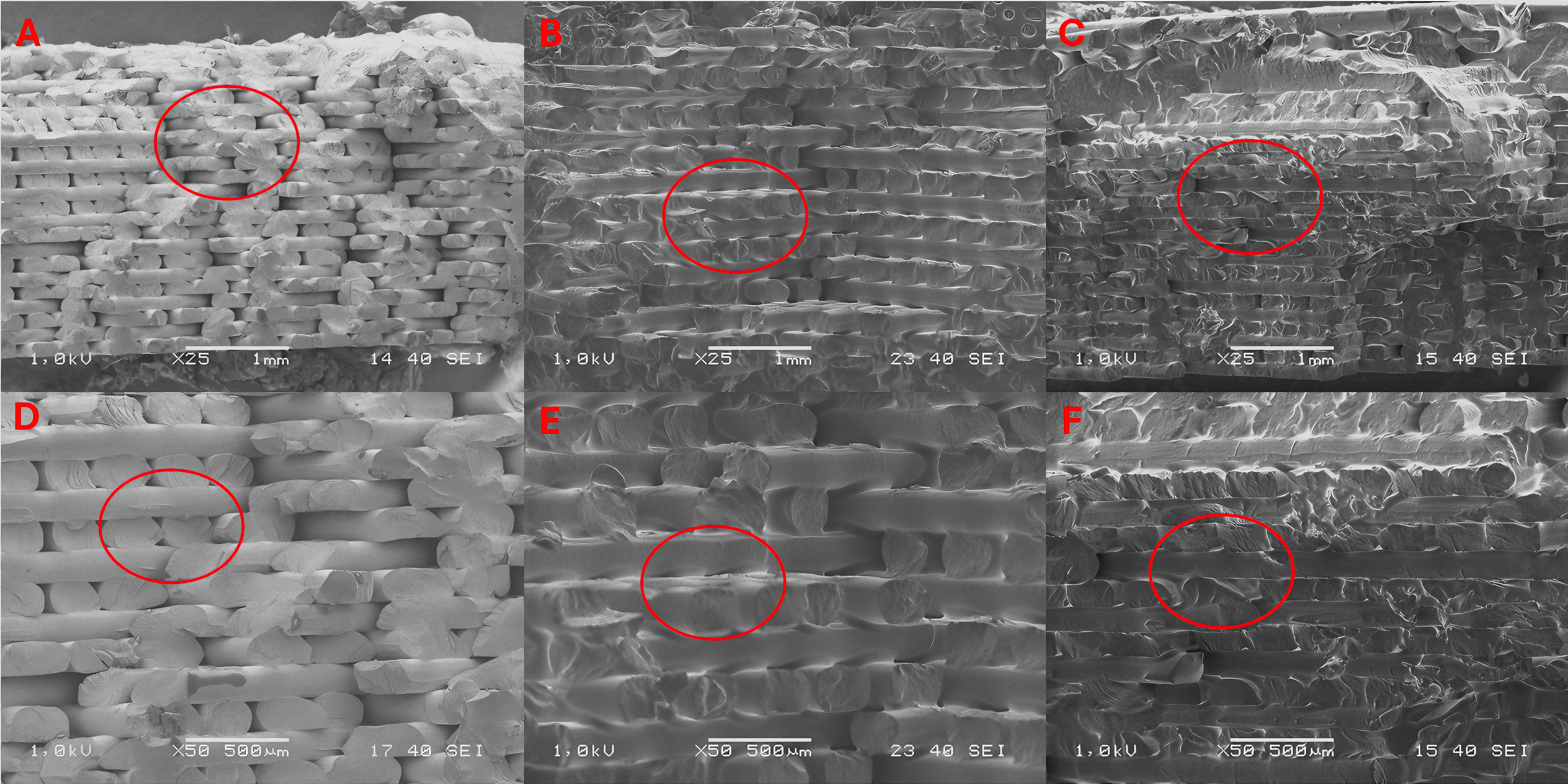

Supplement: Supplementary file 1 [file materials-17-03680-s001.zip › Figure S2.png]

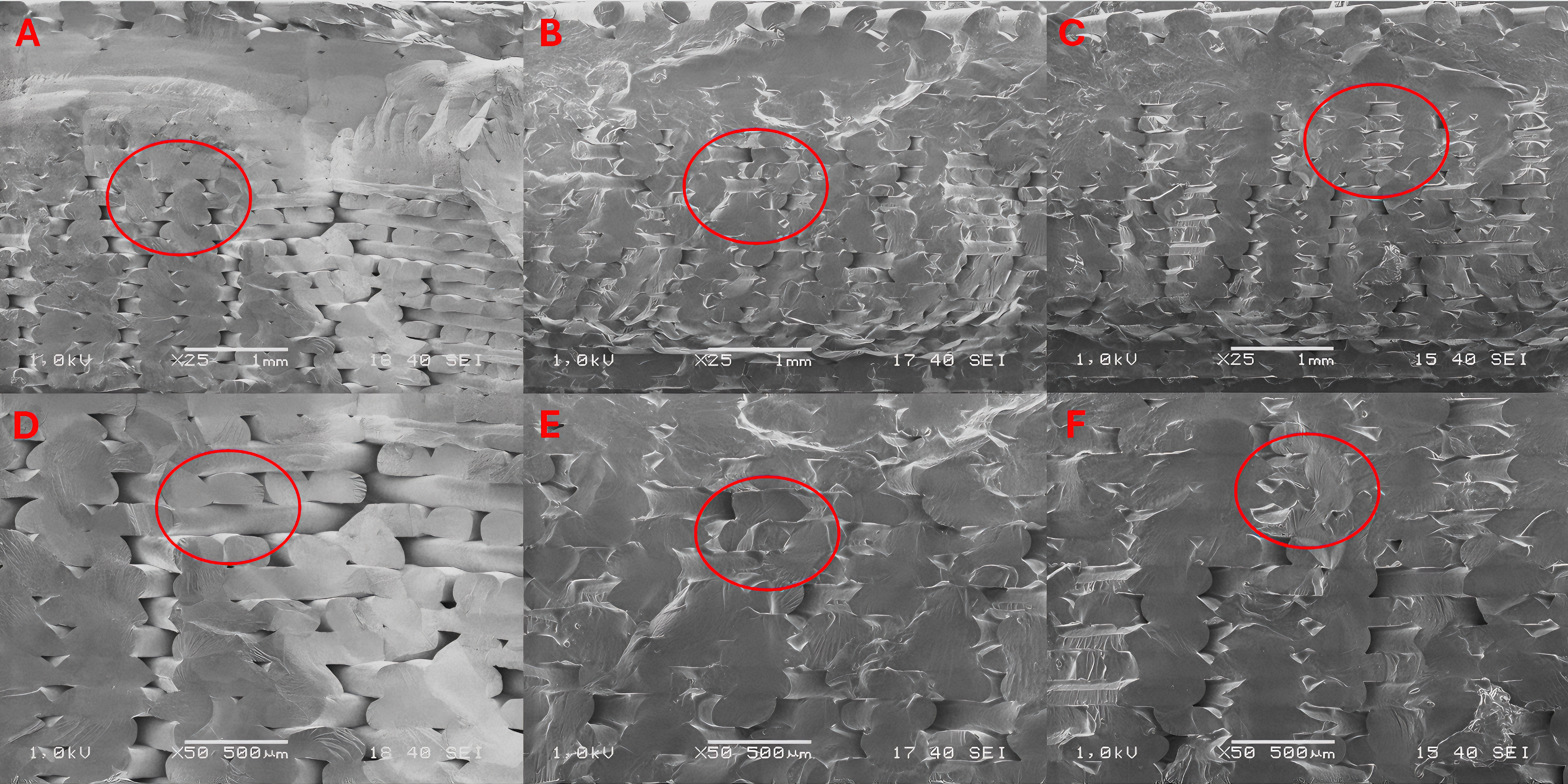

Supplement: Supplementary file 1 [file materials-17-03680-s001.zip › Figure S3.png]

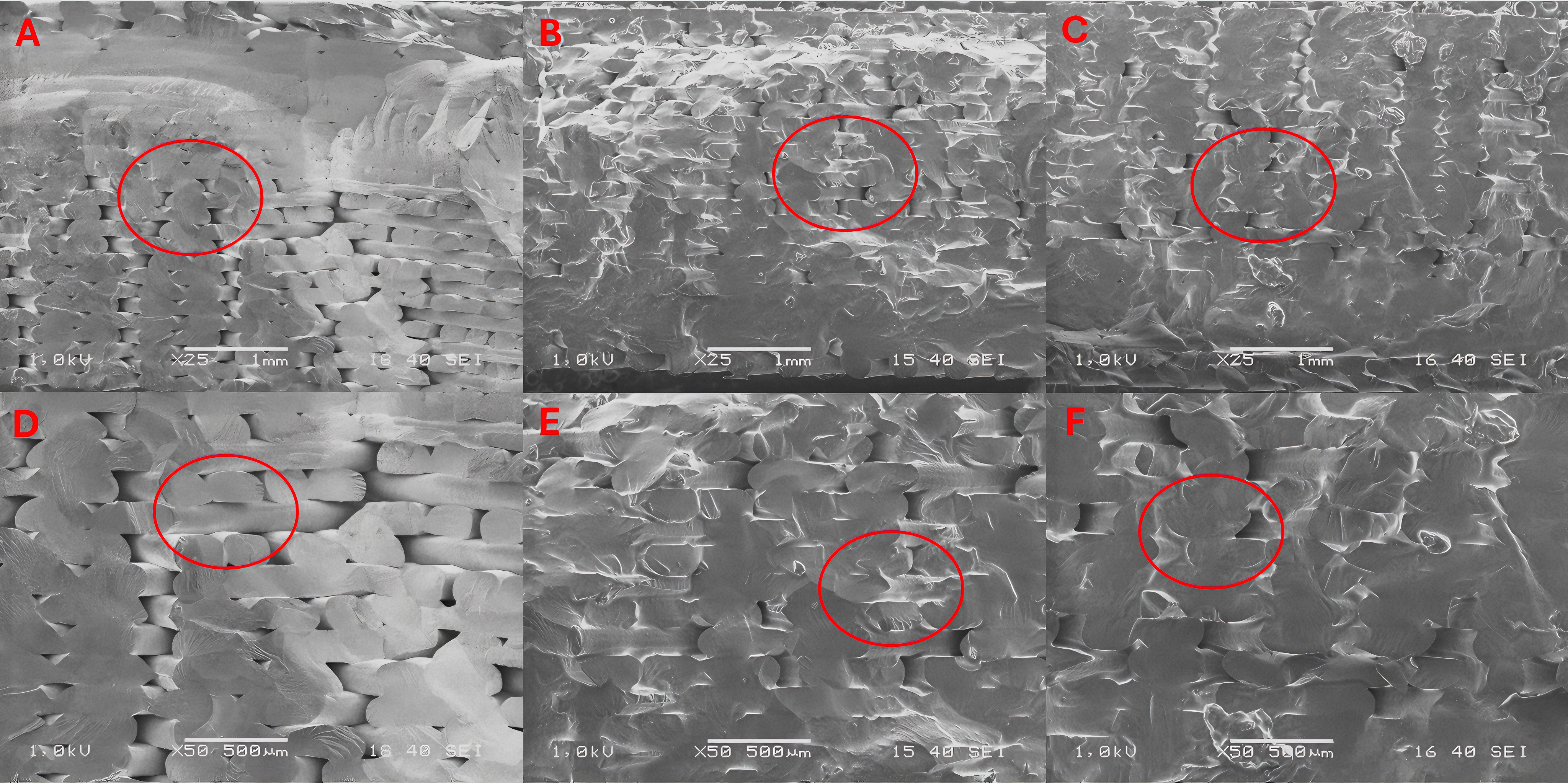

Supplement: Supplementary file 1 [file materials-17-03680-s001.zip › Figure S4.png]

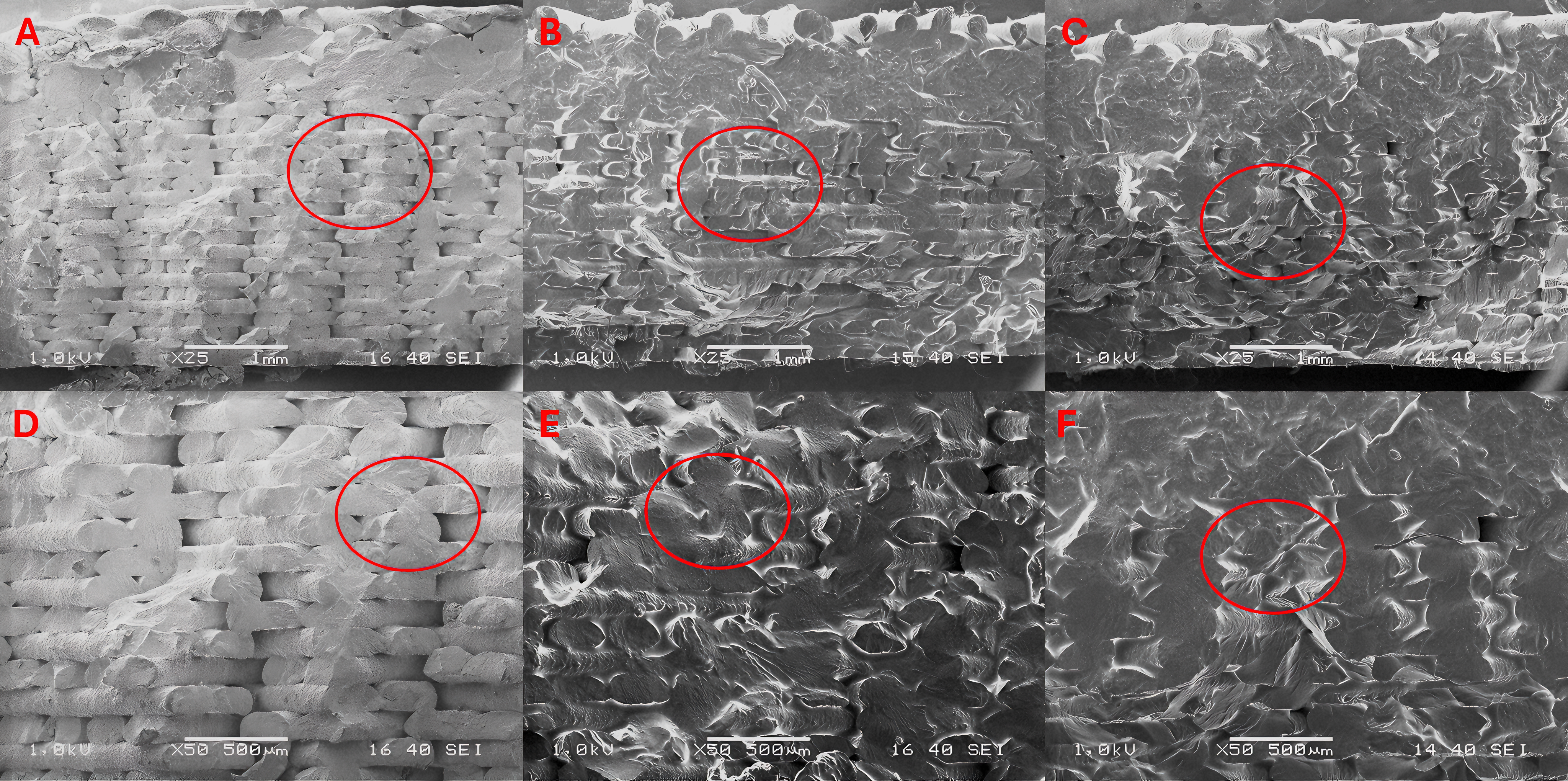

Supplement: Supplementary file 1 [file materials-17-03680-s001.zip › Figure S5.png]

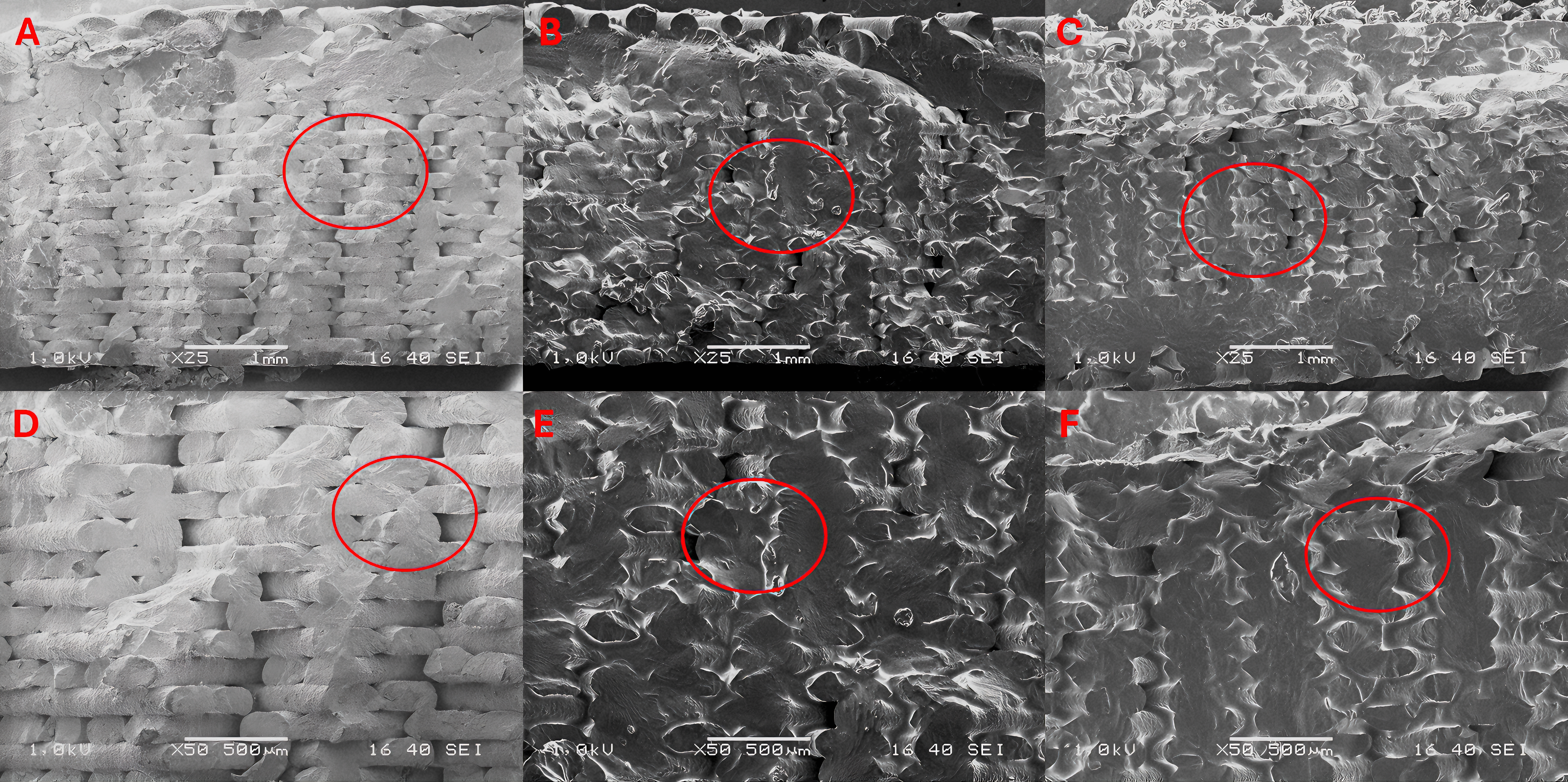

Supplement: Supplementary file 1 [file materials-17-03680-s001.zip › Figure S6.png]

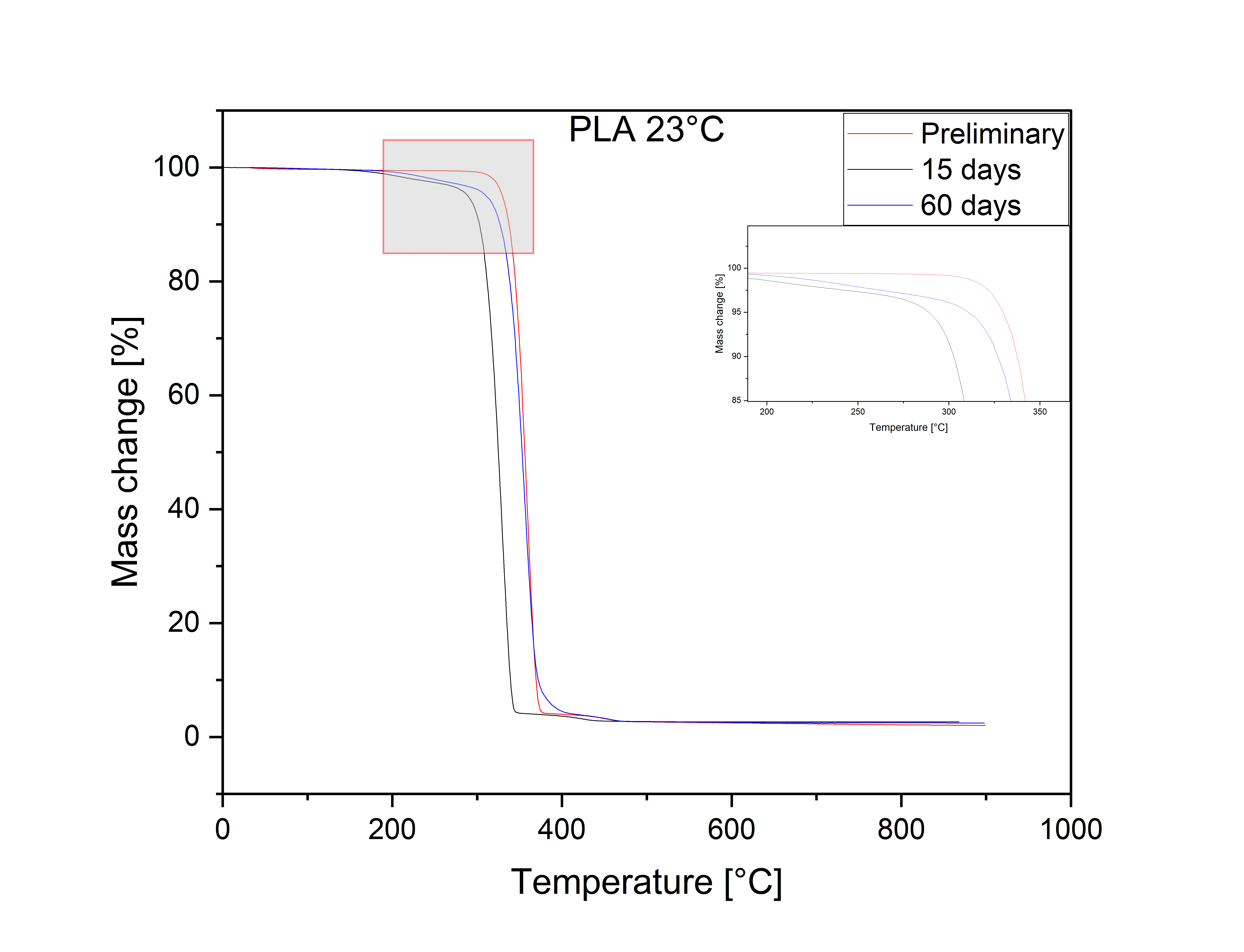

Supplement: Supplementary file 1 [file materials-17-03680-s001.zip › Figure S8.png]

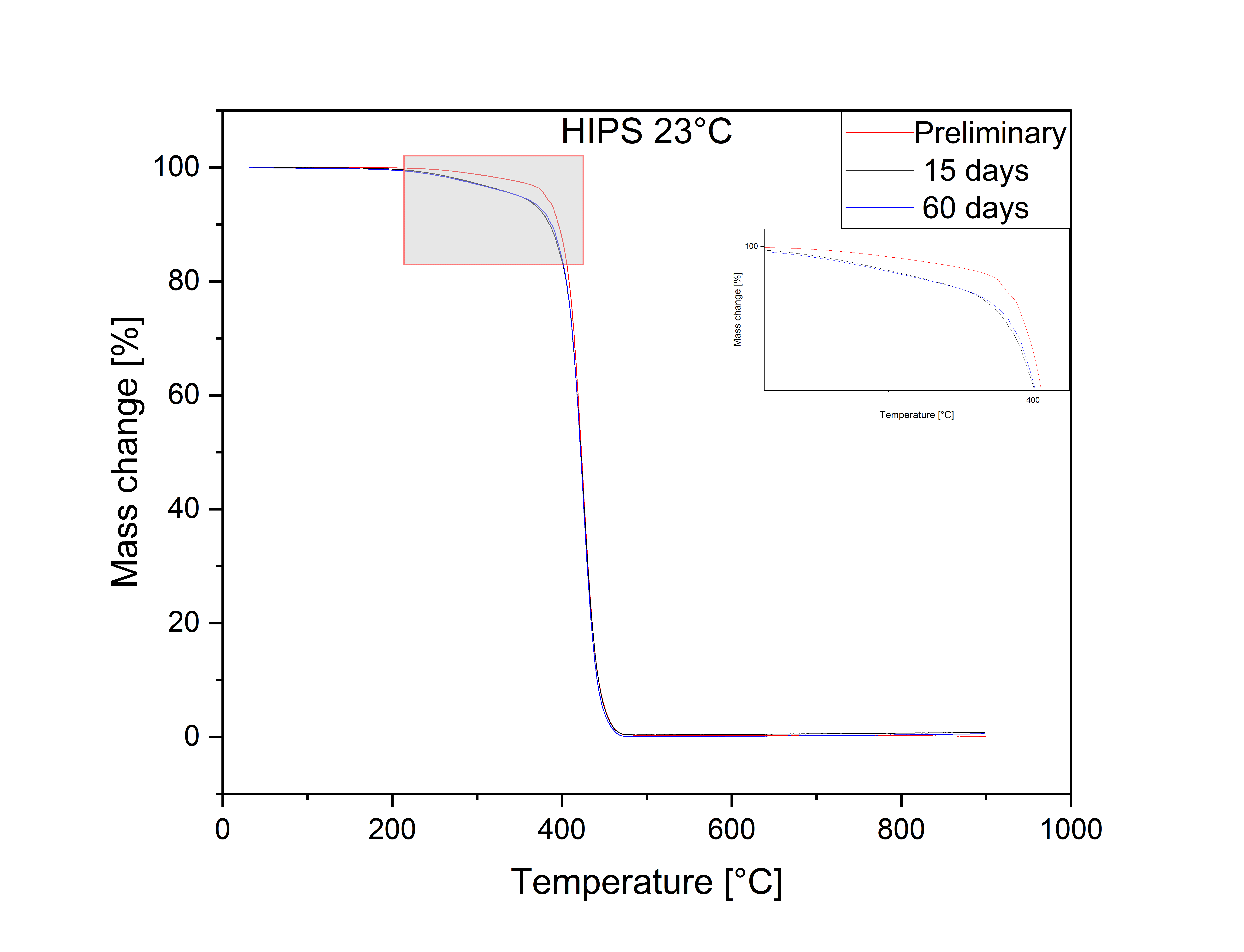

Supplement: Supplementary file 1 [file materials-17-03680-s001.zip › Figure S9.png]
